# Caroli disease with subcutaneous hemorrhage as the sole clinical manifestation: A case report

**DOI:** 10.1097/MD.0000000000036573

**Published:** 2023-12-15

**Authors:** Wentai Yang, Qing Jin

**Affiliations:** a Department of Gastroenterology, First Affiliated Hospital of Gannan Medical University, Ganzhou, Jiangxi Province, China; b Laboratory Medicine, First Affiliated Hospital of Gannan Medical University, Ganzhou, Jiangxi Province, China.

**Keywords:** Caroli disease, liver cirrhosis, subcutaneous hemorrhage, thrombocytopenia

## Abstract

**Rational:**

The disease of Caroli is a rare congenital disorder, characterized by the dilated intrahepatic bile ducts, resulting from mutations in the PKHD1 gene. Caroli syndrome, characterized by dilated intrahepatic bile ducts with congenital hepatic fibrosis, is linked to autosomal recessive polycystic kidney disease. The clinical manifestations of Caroli disease are not typical, and Caroli disease is easy to be missed and misdiagnosed. Therefore, we reported this case in the hope of raising awareness of the disease among clinicians.

**Patient concerns:**

The clinical manifestation of a 10-year-old girl was subcutaneous hemorrhage.

**Diagnoses:**

Magnetic resonance imaging (MRI ) indicates that the person may have Caroli disease, cirrhosis, splenomegaly, portal hypertension, esophagogastric fundal varices, or sponge kidneys.

**Intervention:**

The patient was advised for liver transplantation.

**Outcomes:**

The patient parents did not take our treatment advice, and they asked to go to a better hospital for further treatment, so we did not give the patient any treatment.

**Lessons:**

This case serves as a reminder that if we encounter a patient with hemophilia in our clinic, we should not only consider hematologic diseases and cirrhosis, but also perform an epigastric MRI and magnetic resonance cholangiopancreatography to rule out Caroli disease.

## 1. Introduction

Caroli disease is a rare congenital disorder that is characterized by the congenital diffuse or limited multifocal dilatation of the intrahepatic bile ducts into cystic structures. The cause of the disease remains unknown, and it was first identified by Jacques Caroli in 1958, thus earning its name, Caroli disease.^[1]^ It is widely recognized as an autosomal recessive disorder caused by mutations in the PKHD1 gene,^[2]^ and is the first to describe this condition. It is widely believed to be linked to autosomal recessive polycystic kidney disease. Caroli disease can be diagnosed through the use of ultrasound, computed tomography (CT), MRI, or endoscopic retrograde cholangiopancreatography. MRI is the most precise and noninvasive approach for diagnosing Caroli disease, and it can detect cirrhosis, portal hypertension, and renal abnormalities. Caroli disease encompasses both the simple form, often referred to as Caroli disease, and Caroli syndrome, which is characterized by congenital hepatic fibrosis and/or polycystic kidney disease.^[3]^ The clinical symptoms of patients with Caroli disease can vary greatly, however, in this case, the patient presented with subcutaneous hemorrhage as the only clinical manifestation, which, to my knowledge, is currently the first reported case.

## 2. Case description

**Figure 1.**
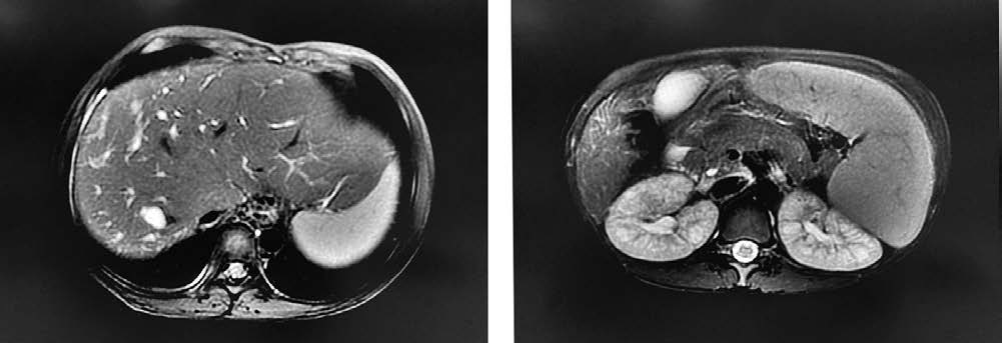
MRI suggested enlarged liver, dilated intrahepatic bile ducts with some distal cystic dilatation, dilated common bile ducts, splenomegaly, increased volume of both kidneys, and mildly dilated fluid accumulation in some of the calyces of both kidneys. MRI = magnetic resonance imaging.

A 10-year-old girl was brought into the hospital with thrombocytopenia that was discovered during physical examination. The child was admitted to the local health center with a diagnosis of an upper respiratory tract infection, and blood tests indicated a possibility of thrombocytopenia, though the exact value was unknown. The child also exhibited petechiae on both lower limbs. The child did not experience any nosebleeds, gum bleeding, fatigue, jaundice, loss of appetite, itchy skin, or abdominal pain. She was hospitalized and diagnosed with thrombocytopenic purpura, acute upper respiratory tract infection, iron deficiency anemia, and splenomegaly. She was discharged after receiving symptomatic supportive treatments such as purging heat and removing toxins, hormonal anti-inflammation, and rehydration. On March 23, 2023, she was once again admitted to a local hospital due to petechiae present on the skin of her lower extremities. Her blood routine revealed a leukocytes count of 3.4 × 10^9^/L, an erythrocytes count of 3.56 × 10^9^/L, and a hemoglobin level of 91 g/L.Hemoglobin level of 91 g/L, platelets of 41 × 10^9^/L, and ultrasound evidence of splenomegaly were observed. The patient has been admitted to our hospital for further diagnosis and treatment, and no other special disease was found. After conducting a physical examination, it was found that the body temperature was 36.6°C.83 beats/min refers to the pulse rate. Respiratory rate of 18 beats/min was recorded. The blood pressure reading was 114/75mmHg. The anemic face indicated a hemoglobin level below normal. There were no obvious abnormalities in the heart and lungs. The abdomen was flat, soft, with no compression pain upon palpation. There was no rebound pain, and the liver was not palpable. However, the spleen could be palpated below the costal margin. Both lower limbs showed no signs of edema. Upon admission, the blood routine indicated a hemoglobin level of 93 g/L, a leukocyte count of 2.82 × 10^9^/L, and a platelet count of 42 × 10^9^/L. The liver and renal function biochemical tests did not reveal any abnormalities, and the results of viral markers such as HBV surface antigen and anti-hcv were negative. The autoimmune hepatopathy antibody was found to be negative, while the copper-cyanin level was normal. The prothrombin time was 13 seconds and the prothrombin activity was 63.8%. MRI of the epigastric area and pancreaticobiliary hydrography indicated the presence of dilatation of both intra- and extrahepatic bile ducts, suggesting Caroli disease, cirrhosis, splenomegaly, portal hypertension, esophagogastric fundal varices, and sponge kidneys (Fig. 1). The patient experienced no gastrointestinal bleeding and symptoms such as abdominal pain and fever. Because the patient had liver cirrhosis, we suggested that the patient should undergo liver transplantation, but the parents thought that the child was too young, did not have serious symptoms, and could not afford the high medical expenses due to financial difficulties. They refused further treatment and asked to go to a better hospital for better treatment. Therefore, we did not administer any treatment and discharged the patient.

## 3. Discussion

Caroli disease is a rare congenital disorder, which is mostly considered autosomal recessive, and has a prevalence of one in a million in the general population.^[3]^ Carole disease may lead to various complications, including cholangitis, sepsis, choledocholithiasis, hepatic abscess, cholangiocarcinoma, portal hypertension, and more.^[4]^ Additionally, symptoms may include right upper abdominal pain, jaundice, and recurrent fever.^[5]^ If cholangitis is present, it can be treated with antibiotic anti-infection therapy, endoscopic retrograde cholangiography stent placement, or percutaneous hepatic puncture for biliary drainage. However, these palliative treatments may lead to recurrent episodes of cholangitis or even cholangiocarcinoma, and many patients die within 5–10 years of the onset of cholangitis.^[4]^ If the lesion is limited, surgical options such as the Roux-en-Y hepaticojejunal anastomosis and various types of hepatectomy can be performed, and recent studies have shown that patients who undergo hepatectomy have a favorable prognosis and longer survival time.^[5]^ Liver transplantation is an effective treatment for Caroli disease and should be considered if liver fibrosis is present.

Caroli disease can develop in childhood or adulthood and has no specific clinical manifestations. Some patients exhibit no discernible clinical symptoms, while others exhibit fever, jaundice, and abdominal pain following physical examination findings. If there are repeated episodes of cholangitis or even intrahepatic abscess and septicemia, the prognosis is poorer. Congenital hepatic fibrosis results in portal hypertension and esophagogastric fundal varices, both of which can cause gastrointestinal hemorrhage.^[6]^ The patient presented no symptoms such as abdominal pain, jaundice, vomiting blood, blood in stool, etc. However, petechiae were observed on the skin of both lower limbs. The primary hospital did not have the necessary equipment to perform an MRI, and thus misdiagnosed thrombocytopenic purpura. Consequently, the patient was admitted to our hospital for further imaging that suggested Caroli disease and spongy kidneys. The patient had cirrhosis and was suffering from portal hypertension. Given the patient young age and the lack of complications such as chronic abdominal pain, cholangitis, sepsis, choledocholithiasis, hepatic abscess, and cholangiocarcinoma, we recommended that the patient undergo liver transplantation. However, the patient family requested further treatment at other hospital after careful consideration.

The limitation of this case is that there are only laboratory and imaging findings and no genetic testing or liver biopsy histology. However, we later followed up with this patient, and her parents stated that she had undergone genetic testing at other hospitals to prove the disease; until now, this patient has not received further treatment.

## 4. Conclusions

Caroli disease is a rare condition that may lead to complications such as cholangitis, gallstones, portal hypertension, and more. If the lesion is limited, a partial hepatectomy may be considered to avoid complications and reduce the risk of malignancy. Additionally, if hepatic fibrosis occurs, liver transplantation may be recommended if the economic situation allows. This case serves as a reminder that if we encounter a patient with hemophilia in our clinic, we should not only consider hematologic diseases and cirrhosis, but also perform an epigastric MRI and magnetic resonance cholangiopancreatography to rule out Caroli disease.

## Author contributions

**Conceptualization:** Wentai Yang.

**Data curation:** Wentai Yang.

**Formal analysis:** Qing Jin.

**Investigation:** Qing Jin.

**Methodology:** Qing Jin.

**Project administration:** Qing Jin.

**Resources:** Wentai Yang.

**Supervision:** Qing Jin.

**Validation:** Wentai Yang.

**Visualization:** Wentai Yang.

**Writing – original draft:** Wentai Yang.

**Writing – review & editing:** Wentai Yang.
